# Delayed specific IgM antibody responses observed among COVID-19 patients with severe progression

**DOI:** 10.1080/22221751.2020.1766382

**Published:** 2020-06-01

**Authors:** Liang Shen, Chunhua Wang, Jianzhong Zhao, Xiaoyong Tang, Ying Shen, Mingqing Lu, Zhe Ding, Canping Huang, Ji Zhang, Shichao Li, Jiaming Lan, Gary Wong, Yufang Zhu

**Affiliations:** a Xiangyang Central Hospital, Affiliated Hospital of Hubei University of Arts and Science, Xiangyang, People’s Republic of China; b CAS Key Laboratory of Molecular Virology & Immunology, Institut Pasteur of Shanghai, Chinese Academy of Sciences, Shanghai, People’s Republic of China; c Department of Microbiology-Infectiology and Immunology, Laval University, Quebec City, Canada

**Keywords:** GICA, delayed, IgM antibody, severity, COVID-19

## Abstract

Severe acute respiratory syndrome coronavirus 2 (SARS-CoV-2) has spread rapidly worldwide since it was confirmed as the causative agent of COVID-19. Molecular diagnosis of the disease is typically performed via nucleic acid-based detection of the virus from swabs, sputum or bronchoalveolar lavage fluid (BALF). However, the positive rate from the commonly used specimens (swabs or sputum) was less than 75%. Immunological assays for SARS-CoV-2 are needed to accurately diagnose COVID-19. Sera were collected from patients or healthy people in a local hospital in Xiangyang, Hubei Province, China. The SARS-CoV-2 specific IgM antibodies were then detected using a SARS-CoV-2 IgM colloidal gold immunochromatographic assay (GICA). Results were analysed in combination with sera collection date and clinical information. The GICA was found to be positive with the detected 82.2% (37/45) of RT-qPCR confirmed COVID-19 cases, as well as 32.0% (8/25) of clinically confirmed, RT-qPCR negative patients (4–14 days after symptom onset). Investigation of IgM-negative, RT-qPCR-positive COVID-19 patients showed that half of them developed severe disease. The GICA was found to be a useful test to complement existing PCR-based assays for confirmation of COVID-19, and a delayed specific IgM antibody response was observed among COVID-19 patients with severe progression.

## Introduction

During December 2019, a cluster of 41 cases of severe viral pneumonia of unknown origin (COVID-19) was reported in Wuhan, Hubei Province, China [1]. A novel coronavirus (SARS-CoV-2), with approximately 80% genome similarity to Severe Acute Respiratory Syndrome Coronavirus (SARS-CoV), was quickly shown to be the causative agent [2, 3]. The outbreak quickly evolved into a pandemic with confirmed cases numbering over 2250,000 and 160,000 deaths as of April 21, 2020 (https://www.who.int/emergencies/diseases/novel-coronavirus-2019/situation-reports/), with cases reported from all 6 permanently inhabited continents. Initial clinical manifestations of the disease include fever, fatigue, and dry cough, while patients with severe disease may exhibit pneumonia and acute respiratory distress syndrome (ARDS) [4]. Full-genome sequencing and phylogenic analysis indicated that SARS-CoV-2 belongs to the betacoronavirus 2b lineage, the same group as Severe Acute Respiratory Syndrome coronavirus (SARS-CoV), another highly virulent pathogens in humans. Bats are suspected to be the reservoir and pangolins are suggested to be an intermediate host for SARS-CoV-2 [5, 6].

Currently, molecular diagnosis of COVID-19 is based on amplification of SARS-CoV-2 RNA extracted from patient respiratory specimens such as nasal swabs, sputum, and bronchoalveolar lavage fluid (BALF) using quantitative reverse transcription polymerase chain reaction (RT-qPCR) [7]. Due to a shortage of diagnostic reagents, some patients can also be clinically diagnosed via chest computed tomography (CT) scan, in which patients show evidence of pneumonia due to the ground-glass opacity (GGO) phenomenon [8]. However, a chest CT scan is not necessarily indicative of COVID-19 as severe infections with other respiratory pathogens can also result in GGO. Therefore, other detection methods especially serology testing specific for SARS-CoV-2 are needed to accurately diagnose of COVID-19 [9].

IgM-based gold immunochromatographic assay (GICA) is a point-of-care testing (POC-T) method for the diagnosis of viral infections in clinical settings [10]. It is known from previous coronavirus studies that two viral structural proteins [spike (S) and nucleocapsid (N)] are involved in the production of IgM antibodies [11]. The S protein is responsible for virion attachment and entry into host cells by binding to its cell receptor and membrane fusion, whereas the N protein is involved in virion assembly, playing a pivotal role in virus transcription and assembly efficiency [12]. Previous studies indicate that both the S and N antigens are involved in the production of specific antibodies and have also shown that IgM responses directed against the N or S antigens can be detected early during days 3–19 of SARS-CoV infection [13–15], but the early antibody response after infection with SARS-CoV-2 infection is currently not well defined.

To investigate the utility of the GICA as a candidate clinical diagnostics assay, we investigated the IgM antibody response in COVID-19 patients in Xiangyang, Hubei Province, China. We show that GICA is a reliable, easy-to-use POC-T method to complement existing nucleic acid-based assays to improve the detection of SARS-CoV-2, and found that a delayed IgM antibody response early during infection significantly correlates with severe disease in COVID-19 patients.

## Methods

### Sample collection

Serum samples were collected from patients at the Xiangyang Central Hospital with informed consent, and the protocols were approved by the hospital's Medical Ethics Committee. Whole blood samples were collected via venipuncture and the sera were separated by centrifugation at 3000×*g* for 20 min within 24 h of collection, in which the supernatant was collected. The sera were then incubated at 56°C for 30 min to inactivate the sample, and stored at −80°C until use.

### Study design

Two studies were performed. In the first study, four panels of human sera (***n* = 130**) were used for the evaluation of the SARS-CoV-2 IgM GICA. Panel A consisted of 45 sera collected from 45 RT-qPCR confirmed COVID-19 cases (range: 4–14 days after symptom onset). Panel B consisted of 25 sera (clinically confirmed but RT-qPCR negative patients, 4–14 days after symptom onset) from 25 patients. Panel C consisted of 10 sera from 10 patients with non-coronaviral respiratory illness (2 confirmed for influenza A virus, 3 confirmed for influenza B virus, 3 confirmed for respiratory syncytial virus and 2 confirmed for adenovirus). Panel D, the negative control, consisted of 50 sera samples collected from 50 healthy people assessed by physical examination during early December 2019, when COVID-19 was not yet reported. For Panel A, 39 patients were classified as mild disease, whereas 6 were classified as severe COVID-19. In the second study, 155 sera samples (from 0 to 31 days after symptom onset) were collected on a longitudinal basis from 58 patients, which included 50 and 8 mild and severe COVID-19 patients, respectively.

### Category for determination between severe vs. mild COVID-19 disease

The severe cases in this study refer to the patients who had enrolled to the intensive care unit (ICU) and received a treatment for more than 3 days, whereas other confirmed cases were distributed to the mild group.

### SARS-CoV-2 IgM GICA

SARS-CoV-2 specific IgM antibodies were detected using the SARS-CoV-2 IgM GICA kit (Shanghai Outdo Biotech Co., China, approved by National Medical Products Administration for SARS-CoV-2, No. 20203400367) following manufacturer instructions. A 15-µl aliquot of the serum was added to the specimen diluent and mixed. Aliquots of 80 µl diluted samples were pipetted into the sample wells of the SARS-CoV-2 IgM GICA cassette and the results were read within 20 min. SARS-CoV-2 IgM antibodies were captured by immobilized SARS-CoV-2 antigen (N and S recombinant proteins forming an antibody–antigen complex) on the test line, in which the result could be detected by anti-human IgM antibodies. The serum was considered positive if bands could be visualized on both the test and control lines. Each sample was repeated in triplicate.

### Statistical analysis

Categorical variables were described as frequency rates and percentages, and continuous variables were described using mean, median, and interquartile range (IQR) values. Means for continuous variables were compared using independent group *t*-tests. Proportions for categorical variables were compared using the *χ*^2^ test, and the Fisher exact test was used instead when the data were limited [16]. All analyses were done with SPSS software and *p*-values of less than 0.05 were considered statistically significant.

## Results

### SARS-CoV-2 IgM GICA is a reliable assay for COVID-19 diagnosis

**Figure 1.**
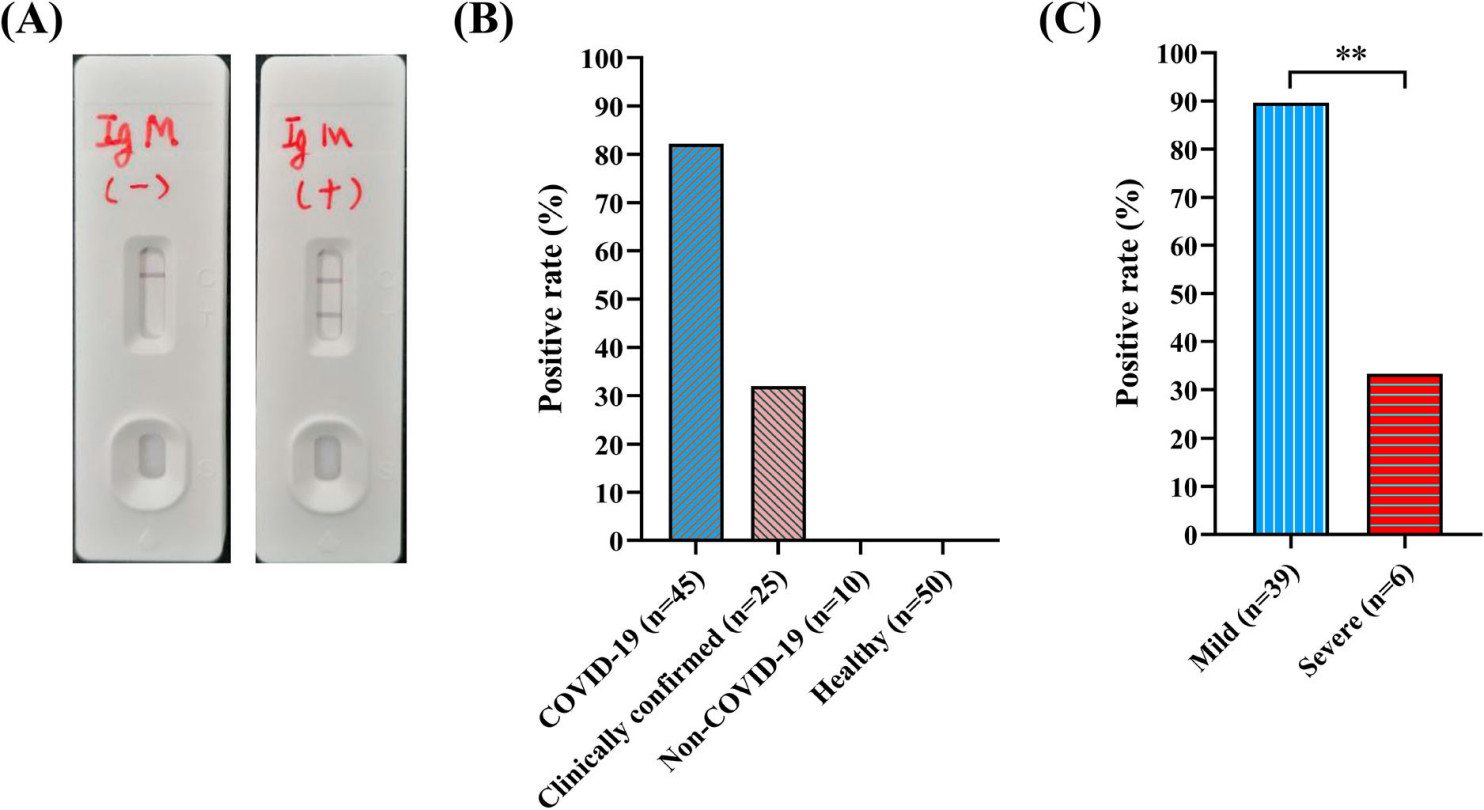
Use of the GICA for the detection of SARS-CoV-2 specific IgM in human sera. (A) Sample negative or positive IgM test. (B) Positive IgM antibody rates between COVID-19 patients, clinically confirmed but RT-qPCR negative patients, non-COVID-19 respiratory illness and healthy people. (C) Comparison of IgM detection rates in RT-qPCR confirmed, mild vs. severe, COVID-19 patients. **P < 0.01.

An example of a negative vs. positive result using the SARS-CoV-2 IgM GICA is shown (Figure 1A). The positive rate of the GICA from the RT-qPCR confirmed COVID-19 patients is shown to be 82.2% (37/45) (Figure 1B), showing that the strip could capture the majority of laboratory-confirmed cases. Interestingly, 32% (8/25) of suspected (clinically confirmed by RT-qPCR negative) COVID-19 patients were also IgM-positive for SARS-CoV-2. The specificity of the GICA was then evaluated using 10 human sera infected with other common respiratory viruses and 50 sera from healthy people. As expected, no SARS-CoV-2 IgM antibodies were detected using the N–S recombinant protein-based GICA assay, indicating that the strip exhibited high specificity. The sample results repeated in triplicate were highly reproducible (data not shown), indicating the reliability of the assay. Since the positive rate of confirmed COVID-19 patient sera collected at 4–14 days after symptom onset was lower than the 100% as detected by RT-qPCR, we sought to understand the reason behind this discrepancy. Preliminary analysis of patient disease outcome appears to suggest that IgM detection rates were substantially lower in those that progressed to severe pneumonia (33.3%, 2/6), as opposed to mild disease (89.7%, 35/39) (Figure 1C). This means that out of the 8 RT-qPCR-positive COVID-19 patients that were GICA-negative for IgM, 50% (4/8) had developed severe disease.

### Igm responses found to be delayed in patients with severe COVID-19

In the second study, we collected 155 samples over time from 58 patients over time (0–31 days after symptom onset). The general information of these patients and the samples collected are shown (Table 1). The median age of these patients is 52 years (IQR, 36–61; range, 8–81 years), and 26 (45%) were men. Of these, 2.8% (2/58) was younger than 18, 27.8% (20/58) was aged 19–40, 47.2% (34/58) was aged 41–65 and 22.2% (16/58) was older than 65. From these patients, 13.8% (8/58) progressed to severe COVID-19. There was found to be a significant difference in the age of severe patients (median age, 68 years) compared to mild patients (median age, 49 years) (*p* < 0.01). A total of 121 samples were collected for 50 mild patients and 34 samples for 8 severe patients (Table 1).

**Table 1.** Clinical characteristics and sera of patients with COVID-19 (*n* = 58).

Characteristics	Total (*n* = 58)	Mild (*n* = 50)	Severe (*n* = 8)	*p*-Value^a^
Age: Median (IQR)	52 (36–61)	49 (35–58)	68 (57–77)	<0.01
Age groups: *n* (%)				
≤18	2 (2.8)	2 (4.0)	0 (0)	
19–40	20 (27.8)	16 (32.0)	0 (0)	
41–65	34 (47.2)	23 (46.0)	4 (50.0)	
≥66	16 (22.2)	9 (18.0)	4 (50.0)	
Gender: *n* (%)				
Female	32 (55)	27 (54.0)	5 (62.5)	0.72
Male	26 (45)	23 (46.0)	3 (37.5)	
Sera: *n* (%)	Total (*n* = 155)	Mild (*n* = 121)	Severe (*n* = 34)	
Less than 4 days after symptom onset	41 (26.5)	34 (28.1)	7 (20.6)	0.39
4–7 days after symptom onset	31 (20.0)	25 (20.7)	6 (17.6)	
8–14 days after symptom onset	48 (31.0)	39 (32.2)	9 (26.5)	
15–21 days after symptom onset	23 (14.8)	15 (12.4)	8 (23.5)	
More than 21 days after symptom onset	12 (7.7)	8 (6.6)	4 (1.8)	

Abbreviations: IQR, interquartile range.

^a^*p*-values indicate differences between mild and severe patients. *p* < 0.05 is considered statistically significant.

**Figure 2.**
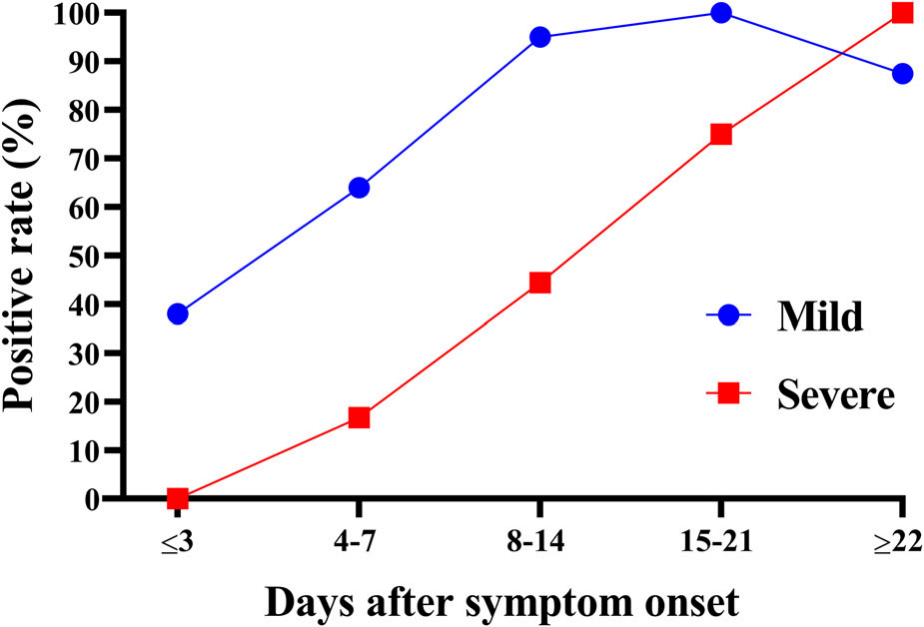
IgM responses in mild vs. severe COVID-19 patients. (A) The positive rates of the SARS-CoV-2 IgM antibodies in mild and severe COVID-19 patients over time. *P* < 0.01.

The positive rates of SARS-CoV-2 IgM antibodies were found to increase gradually over time in the sera of both mild and severe patients and significant differences can be observed (*p* < 0.01). We observed that the positive rate in the mild group was 38% (13/34), compared to 0% (0/7) in the severe group at ≤3 days after symptom onset (Figure 2). At 4–7 days after symptom onset, the positive rate of the mild group was 64% (16/25), compared to 16.7% (1/6) in the severe group (Figure 2). At 15–21 days after symptom onset, the positive rate of the mild group was 100% (15/15), compared to 75% (6/8) in the severe group (Figure 2). These results suggest that the development of detectable levels of SARS-CoV-2 IgM antibodies in severe COVID-19 patients is delayed. To provide a more intuitive observation of the changes and rise of the SARS-CoV-2 IgM antibodies over time in mild vs. severe patients, we provide a table summarizing the GICA results of 7 patients with over 5 sera samples collected for each subject (Table 2). It can be observed that IgM could be observed in mild patients (P1–P3) by latest 6 days after symptom onset, but the earliest time point that IgM could be observed in severe patients (P4 to P7) is at 8 days after symptom onset.

**Table 2.** Development of the specific IgM responses over time in seven patients with COVID-19.

Patients		Days after illness onset																							
		1	2	3	4	5	6	7	8	9	10	11	12	13	14	15	16	17	18	19	20	21	22	23	24
Mild	P1			+		+				+						+				+					
	P2		–		+						+							+							+
	P3				–		+						+					+					+		
Severe	P4			–	–				–		+		+					+							
	P5						–						–			–	–	+							
	P6	–		–				–			–					+					+				
	P7						–		+		+								+					+	

## Discussion

For effective prevention, control and treatment of infectious diseases, an early and accurate diagnosis of cases are crucial. Nucleic acid-based detection of SARS-CoV-2 by RT-qPCR from nasal or throat swabs, the sputum or BALF samples is currently the favoured diagnostic method for COVID-19 [17]. However, it has been reported by several groups that nasal or throat swabs do not necessarily yield high detection rates from infected patients (or in other words, false negatives) [18], patients do not always produce sputum during disease, and collection of BALF samples pose a problem as it is dangerous and difficult for medical staff to obtain, and painful for the patient. In addition, sample quality and preservation can also be difficult when the medical system is overwhelmed, resulting in substantial and unwanted differences of diagnostic results. While viremia has rarely been observed in COVID-19 patients [19] and thus there is limited utility in testing blood samples by RT-qPCR, specific IgM typically arises within days after infection. Thus, in this study, we tested the use of an easy-to-use, clinically compatible GICA assay for diagnosis of COVID-19 from patient sera, which is a simple and standard procedure for medical professionals working in the hospital.

According to previous studies, recombinant S and N proteins in combination as detection antigens can improve detection rates against MERS-CoV and SARS-CoV [14]. The GICA used in this study employs the same strategy of N–S recombinant protein as capture antigens for SARS-CoV-2 IgM antibodies. As shown in the results, GICA can detect the majority of RT-qPCR confirmed COVID-19 patients, whereas a proportion of clinically confirmed COVID-19 cases were also confirmed with this method. Thus the GICA can be used as supplementary complement to nucleic acid-based and chest CT tests to improve the positive detection rate of COVID-19 patients.

Our preliminary analysis of the remaining missed IgM tests in the RT-qPCR confirmed samples led us to surmise that severe COVID-19 patients do not develop detectable levels of IgM antibodies early on in disease. Indeed, our longitudinal analysis of patient sera in 50 mild vs. 8 severe cases show that there is a significant decrease in the detection rates of SARS-CoV-2 IgM early after symptom onset, from days 4 to 14. While the positive rate of sera in the mild group declined to 87.5% (7/8) when sampled at longer than 21 days after symptom onset (Figure 2), this is expected as IgM level are known to decline as IgG levels rise. The IgM-negative serum sample (which was identified to be collected at 30 days after symptom onset) was further tested using a combined GICA assay (IgM + IgG) using recombinant S and N proteins in combination as detection antigens, and the result was found to be positive (data not shown).

A potential drawback in our study is that due to current technical and manpower limitations we did not quantitate and compare the endpoint titers of IgM in the mild vs. severe patients by ELISA, and thus we do not know what the limits of detection of these antibody-based assays may be. However, our data shows that the GICA is a useful complementary assay for the diagnosis of COVID-19, and demonstrated that a delayed production of SARS-CoV-2 IgM antibody response based GICA could be an indicator for the severity of COVID-19 in patients. It has been reported that lymphopenia is commonly observed in COVID-19 patients [20], which may partially explain for our observations in the clinic. Future studies should explore the decreased lymphocyte types by flow cytometry and investigate the molecular mechanisms of lymphocytopenia from the aspect of virus-host interactions, and suppression of natural immune by viral coding proteins [21–23], and to confirm our findings in this study with larger patient sample sizes.
